# Using deep belief network modelling to characterize differences in brain morphometry in schizophrenia

**DOI:** 10.1038/srep38897

**Published:** 2016-12-12

**Authors:** Walter H. L. Pinaya, Ary Gadelha, Orla M. Doyle, Cristiano Noto, André Zugman, Quirino Cordeiro, Andrea P. Jackowski, Rodrigo A. Bressan, João R. Sato

**Affiliations:** 1 Center of Mathematics, Computation, and Cognition. Universidade Federal do ABC, Santo André, Brazil; 2 Department of Psychiatry. Universidade Federal de São Paulo, São Paulo, Brazil; 3 Department of Neuroimaging, Institute of Psychiatry, Psychology and Neuroscience, King’s College London, London, United Kingdom; 4 Interdisciplinary Lab for Clinical Neurosciences (LiNC), Universidade Federal de Sao Paulo, Sao Paulo, Brazil; 5 Department of Psychiatry, Faculdade de Ciências Médicas da Santa Casa de São Paulo, São Paulo, Brazil

## Abstract

Neuroimaging-based models contribute to increasing our understanding of schizophrenia pathophysiology and can reveal the underlying characteristics of this and other clinical conditions. However, the considerable variability in reported neuroimaging results mirrors the heterogeneity of the disorder. Machine learning methods capable of representing invariant features could circumvent this problem. In this structural MRI study, we trained a deep learning model known as deep belief network (DBN) to extract features from brain morphometry data and investigated its performance in discriminating between healthy controls (N = 83) and patients with schizophrenia (N = 143). We further analysed performance in classifying patients with a first-episode psychosis (N = 32). The DBN highlighted differences between classes, especially in the frontal, temporal, parietal, and insular cortices, and in some subcortical regions, including the corpus callosum, putamen, and cerebellum. The DBN was slightly more accurate as a classifier (accuracy = 73.6%) than the support vector machine (accuracy = 68.1%). Finally, the error rate of the DBN in classifying first-episode patients was 56.3%, indicating that the representations learned from patients with schizophrenia and healthy controls were not suitable to define these patients. Our data suggest that deep learning could improve our understanding of psychiatric disorders such as schizophrenia by improving neuromorphometric analyses.

Schizophrenia (SCZ) is a complex psychiatric disorder characterized by abnormal brain function, including cognitive deterioration, aberrant sensory perception and disturbed thinking^1^. Neuroimaging-based models aim to improve our understanding of SCZ’s pathophysiology, by revealing and quantifying the anatomical characteristics of the brain relevant to clinical conditions. However, the diagnosis of SCZ is still difficult due to its heterogeneity, the blurred boundary with other major psychiatric disorders, and the lack of objective biologically-grounded measures in clinical practice^2^,^3^. A diagnostic system aided by neuroimaging data would allow for a more objective approach, as well as potentially increasing diagnostic sensitivity and our ability to predict an individual’s prognosis and treatment response^4^.

Several findings of abnormal brain morphometry in SCZ have been observed in magnetic resonance (MR) images, including reduced cortical thickness and subcortical volume^5^. However, there is a high level of variability in these results possibly reflective of the heterogeneity of the disorder. This limits the creation of empirically grounded hypotheses and their proper evaluation. Machine learning methods are capable of representing latent (invariant) features of brain structure, allowing for better representation of SCZ-related processes. However, conventional machine learning models with “shallow” architecture, e.g. support vector machines (SVMs), cannot adequately capture this type of complex information. For this reason, more robust pattern classification models with a hierarchical multilayer structure could be a promising alternative to the shallower image-based prediction methods.

Deep learning is a relatively new field in machine learning^6^,^7^. Its methods automatically create multilevel models with hierarchical representations of the input data. The higher-level representations of the model correspond to abstract concepts, defined therein as a non-linear composition of lower-level representations. For classification tasks, these abstract representations are more resistant to the irrelevant variations (e.g. noise) that are frequently present in the input data. These abstract features amplify the various explanatory factors that are important for discrimination^7^. Recently, deep learning methods have been introduced to medical image analysis with promising results in multiple applications, including computerized prognosis for Alzheimer’s disease^8^,^9^,^10^, tumor segmentation^11^, and histopathological diagnosis^12^,^13^. Neuroimaging analysis is frequently utilized for increasing diagnostic ability, although some studies are using deep learning models to discover the diverse patterns in patient data characteristic of a disease^14^,^15^,^16^,^17^. Accordingly, this multivariate analysis on the latent features can potentially address the issue of high variation across subjects, and then verify the most invariant, abstract features within the input data. This approach is important in enhancing transparency (compared to “black box” models), enabling the level of trust needed for its application in clinical practice.

The deep belief network (DBN)^6^ model is a deep learning model that has gained in popularity as a successful implementation of an efficient learning technique that stacks simpler models known as restricted Boltzmann machines (RBMs)^6^. This unsupervised learning builds amultilevel structure layer-by-layer, automatically extracting increasingly more abstract representations from the layers. After this process, the DBN can then be used to initialize the weights between adjacent layers of a deep neural network (DBN-DNN). By this process, the DBN-DNN mostly avoids the vanishing gradient problem that can occur when training a standard neural network (without pre-initialization). DBN-DNN pre-training also improves model performance by avoiding overfitting and enhancing the model generalisation. This point is critical to neuroimaging analyses given the limited number of samples available in psychiatric neuroimaging. Finally, a supervised fine-tuning process trains the whole DBN-DNN, producing a multilayer model able to perform the desired task (e.g., classification). With such architecture and training, feature selection and extraction can be systematically carried out with no need of explicit ad-hoc elaborations. Another advantage of the DBN is that it is a generative model, and as such can generate samples based on the features that the model learns during training. Sampling the various representations of the clinical condition from the DBN can verify what the model considered important while it was creating the hierarchical structure of features from the input data.

While DBN is extensively used for image recognition and speech processing, only a few studies have applied it to the complex data of differences in brain morphometry related to psychiatric disorders^18^,^19^. Plis *et al*.^18^ demonstrated several examples of the application of deep learning methods to functional and structural brain imaging data. Their results showed the high potential of exploratory analysis with DBNs for learning the physiological representations and detecting the latent relationships in neuroimaging data acquired from patients with Huntington’s disease and SCZ. Kuang and He^19^ applied the DBN to feature extraction and classification tasks in fMRI data acquired as part of the ADHD-200 cohort^20^. The model was found to be useful in discriminating patients with attention deficit/hyperactivity disorder (ADHD) from controls, and it performed somewhat better than the performance described in the results published at the ADHD-200 competition.

In this study, we investigated the use of the DBN to explore and extract latent features from brain morphometry data from healthy controls (HC) and patients with SCZ. We first trained the DBN-DNN and compared its classifier performance to the linear SVM classification algorithm, a widely used shallow-structure machine learning method. Then high-level representations were generated to analyse the differences in the latent features between the two groups. Multivariate analysis was then used to visualize the brain regions most affected by the disease. Finally, we evaluated the use of the trained classifier to predict the diagnosis of subjects in the early stages of the disease.

## Methods

### Subjects

One hundred and forty-three patients with SCZ were recruited from an outpatient unit (Schizophrenia Program - PROESQ) at the Universidade Federal de São Paulo (UNIFESP). The diagnosis was made by trained psychiatrists, according to the DSM-IV criteria using the Structured Clinical Interview for DSM-IV (SCID-I)^21^. The duration of illness was defined as the difference in years between age at onset and age at the investigation. Eighty-three HC were recruited from a governmental employment agency. They had no personal history of psychiatric disorders, nor did they have first-degree relatives with a lifetime history of psychotic illness. Finally, thirty-two patients with first-episode psychosis (FEP) were recruited from a psychiatric emergency unit at the Irmandade da Santa Casa de Misericórdia de São Paulo (ISCMSP) (demographic data in Table 1).

No subject had a history of neurological illness or traumatic brain injury with loss of conscience. All participants provided written informed consent and the study was approved by the Local Ethics Committee of UNIFESP and ISCMSP. The methods of this study were carried out in accordance with relevant guidelines and regulations.

### MRI acquisition

All imaging data were collected at the Department of Imaging Diagnosis of UNIFESP on a 1.5 T Siemens MRI system (Magnetom Sonata A.G.; Siemens Medical Solutions, Erlangen, Germany) with an 8-channel head coil (Siemens, MAGNETON Sonata). A 3DSPGR pulse sequence was used to obtain a T1 anatomical brain image of each subject (up to 192 slices for whole brain coverage; 1.0 mm slice thickness; TE = 3.42 ms; TR = 2000 ms; 15° flip angle; 245 mm FoV; 256 × 256 matrix; number of excitations = 1). In cases of partial brain coverage, the voxel size was allowed to increase up to 0.15 mm.

### MRI processing

FreeSurfer pipeline (v5.0) was applied to the MRI scans to estimate the cortical thickness and anatomical structure volumes. This estimation was performed using the “recon-all” command (more detailed information about the processing in refs 22, 23, 24). The cortical surface of each hemisphere was parcellated according to the Desikan-Killiany atlas^25^. This process calculated the cortical thickness for each of the sixty-eight brain regions (thirty-four in each hemisphere) and volumes of the forty-five anatomical structures (saved as stats/aseg.stats under the FreeSurfer subject directory).

We applied a multiple linear regression on each morphometric feature to reduce the effect of confounders (age and gender). In this process, the variables age and gender were defined as an independent variable, and each morphometric feature was the dependent variable. The resulting residuals were used as corrected input data. Finally, we performed a Z-score normalisation across all subjects in each residual to improve the convergence speed and the stability during the model training. These normalised values of each brain region were used in subsequent analyses.

### Deep learning model

**Figure 1:**
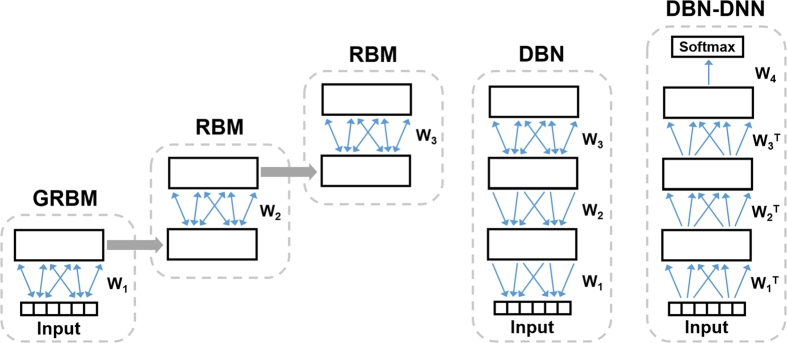
A deep neural network pre-trained by a deep belief network (DBN-DNN). The sequence of steps to create a DBN using greedy-layer-wise pre-training and convert it to a DBN-DNN. First, a Gaussian restricted Boltzmann machine (GRBM) has its weights (W1) optimised to represents in its hidden binary units the distribution of the input data. After this training, the weights are frozen, and the input data are propagated through them to generate a level 1 output. This output is used to train a restricted Boltzmann machine (RBM). The RBM weights (W2) are frozen too, and a third and final RBM has its weights (W3) optimised to represent the correlation between its visible units. All these weights are combined in one structure called a DBN. Finally, a softmax layer is added to the top of the DBN, and all the layers undergo supervised fine-tuned as one DNN.

We implemented our classifier using a deep neural network initialized by a DBN (DBN-DNN). The training of a DBN-DNN is performed in two steps: DBN pre-training and supervised fine-tuning (Fig. 1).

The pre-training corresponds to an efficient learning technique that stacks RBMs^6^, which are independently trained layer-by-layer. Briefly, an RBM adjusts its parameters such that the probability distribution represented by it fits the distribution of the training data as well as possible. At the beginning of DBN pre-training, an RBM that can handle continuous data distribution (known as a Gaussian RBM) learns how to represent the input data distribution from the training examples. After the weights have been learned by this first RBM, the RBM is applied to each training sample and the probability for activating its hidden layers can be calculated and used as the visible layer for another RBM. This method of training an RBM can be repeated several times to create a multilayer model. In this study, the training of each RBM was carried out using contrastive divergence algorithm with 1 alternating Gibbs sampling step. At the beginning of learning, the weights are initialized to values sampled from a Gaussian distribution with mean 0 and standard deviation of about 0.01. The visible and hidden biases are initialized to 0^26^. The learning process was performed with a mini-batch size of 10 samples and was completed over ten epochs.

The pre-trained network is then refined by supervised training that fine-tunes all layers jointly to perform the classification task. This fine-tuning is done by initiating the parameters of a deep neural network with the values of pre-trained DBN parameters. After that, a final layer composed of two softmax units to perform a binary classification is added to implement the desired targets of the training data, the labels SCZ and HC. Afterward, the backpropagation algorithm and a gradient-based optimisation algorithm can be used to adjust the network parameters, creating a DBN-DNN. In this study, the DBN-DNN was trained using a stochastic gradient descent algorithm with the technique of momentum of Nesterov combined with the RMSPROP method^26^,^27^. The maximum value set for the momentum term was 0.9 and a L2 parameter norm penalty (or weight decay) of 5*10^−5^ was applied. This gradient-based optimisation was completed over 200 training epochs, and the mini-batch size was set to 10 training examples. Full details on the theory related to DBNs is provided in the supplementary information. In this study, the DBN-DNN classifiers were developed using the Theano-based^28^ library called pydeeplearn (available at https://github.com/mihaelacr/pydeeplearn).

### Selecting the optimal DBN-DNN

In this study, we verified the effect of the number of layers in the DBN-DNN on classifier performance. We tuned the structure of the DBN-DNN by fixing the number of hidden layers to 1, 2, 3, 4 or 5, where the hidden layers had the same number of units, as suggested by Larochelle *et al*.^29^. To ensure the best performance and reproducibility, the number of neurons per layer, the pre-training learning rate, and the fine-tuning learning rate were calculated through automatic Bayesian optimisation. Table 2 shows the hyperparameters search spaces.

The Bayesian optimisation is a process that generates a set of hyperparameter values, trying to get a better performance from the DBN-DNN classifier based on the achievements of past attempts^30^. In this study, the optimisation process was performed for 2000 iterations for each structure with each different number of layers. The optimisation algorithm consisted of a mixed use of the Tree-of-Parzen-Estimators algorithm (used in 70% of the iterations), the Annealing algorithm (20% of the iterations), and Random Search (10% of the iterations). The optimisation process had the goal of maximizing the mean area under the curve (AUC-ROC) carried out by the classifier on the validation set using a 3-fold cross-validation process (explained in details in Section “Classifiers evaluation”). The Bayesian optimisation was implemented using the Hyperopt library^31^.

### Support Vector machines

We used the SVM classifier as a reference to evaluate our deep learning method. Several studies have already used it to examine the diagnostic and prognostic potential of neuroimaging in a range of psychiatric disorders^32^,^33^. In this study, we chose a linear kernel to train the two-class SVM using the implementation based on libsvm^34^ from the Scikit-Learn library^35^. No feature extraction and feature selection were performed, in order to keep the same input data from the deep learning approach. The soft margin parameter (C) that controls the trade-off between having zero training errors and allowing misclassifications was optimised using the training data and the validation data via a grid search (i.e., C = 0.001, 0.1, 1, 10, 100, and 1000). The measure used during the grid search was the average of the AUC-ROC of the classifier obtained from the validation sets using the same 3-fold cross-validation process and the same training sets of the DBN-DNN optimisation process.

### Classifiers evaluation

**Figure 2:**
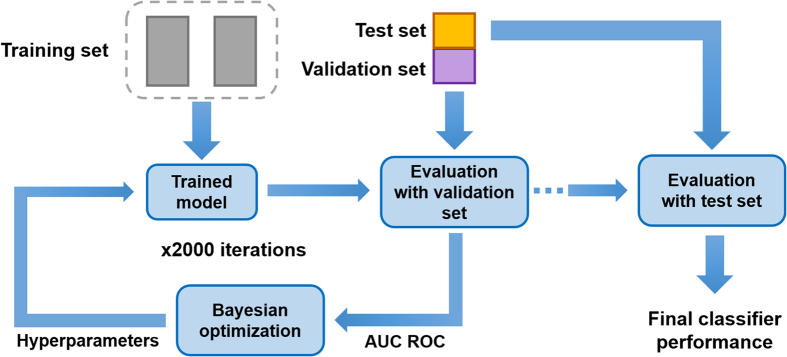
Diagram of the hyperparameter optimisation process and cross-validation. Each rectangle represents a fold of the 3-fold cross-validation. Two folds are used for classifier training, and the third fold is divided into two sets: test and validation. The trained classifier model is evaluated by predicting the classes of the samples of the validation set. The performance measure used in this assessment is the area under the ROC curve (AUC-ROC). This measure is used in the optimisation process of hyperparameters, and new values are created for the next iteration. This optimisation process consists of 2000 iterations of this classifier training and evaluation process. After the optimisation of the hyperparameters, the classifier was trained again with the best hyperparameter values, and then evaluated using the test set.

To determine the performance of the DBN-based classifier in predicting whether a subject belongs to the SCZ group (Fig. 2), the entire dataset was first shuffled and divided into three folds to perform a 3-fold cross-validation. Two folds were used as training set, and the final fold was randomly split into two groups: the validation set and the test set. The validation group is used throughout the Bayesian optimisation while the test samples are used only to obtain the final classifier performance.

During Bayesian optimisation, the DBN-DNN was trained with the training set, and the AUC-ROC was calculated on the validation set. In the following optimisation iterations, the optimizer generates a new set of hyperparameters within their defined search space. The values choice was made based on the performance of past iterations. After 2000 iterations, the DBN-DNN classifier with the hyperparameter values that reached the higher AUC-ROC was used to predict the label of the subjects from the test set.

From these predictions, we computed the following four performance indicators to compare the performance indicators across the machine learning methods.


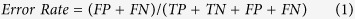



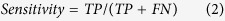



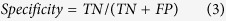



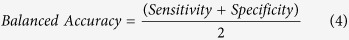


where TP is the number of the true positives, TN is the number of true negatives, FP is the number of false positives, and FN is the number of false negatives.

For the robustness to data dependence, we executed the 3-fold cross validations five times. In each cross-validation process, the partition of training and testing data is randomly determined. The average of these five performance measures was the final classifier score.

In the evaluation of the SVM classifier, we used the same training and test sets used by the deep learning model. The average of the five replications of the 3-fold cross validation was defined as the final classifier score.

### Sampling DBN-DNN

One of the main objectives of this study was the investigation of the most abstract features that are invariant across the patient with SCZ, learned using a multivariate analysis of the latent features of the DBN-DNN. Therefore, we investigated the learned representations of the DBN-DNN model with the highest AUC-ROC assessing on the validation set. Since the DBN is a generative model, it can generate samples from the learned labels. To obtain these samples, we performed 1000 iterations of alternating Gibbs sampling in the top-level RBM (as described in Hinton *et al*.^6^). During the Gibbs sampling, we clamped the label units to a particular class. This approach allows the visualisation of the representation of the model’s class-conditional distributions. We used the sample from this distribution as input to the layers below and generated an input vector (neuromorphometric data) by a single down-pass through the generative connections. This DBN-DNN sampling was performed for each class: SCZ and HC. We calculated the difference between these input vectors to analyse the brain region features learned by the DBN-DNN across the SCZ and HC samples, in order to identify those which best differentiated the classes.

### Analysis of patients with first-episode psychosis

Finally, the trained DBN-DNNs with the optimal number of layers were used to classify subjects in the FEP stage. We measured classifier performance by the error rate, where the classifier erroneously defined the patient with FEP as a healthy control. This analysis was utilised to assess whether the trained DBN-DNN with chronic SCZ has the necessary features to identify this early stage rather to evaluate the classifier performance.

## Results

### Classification performance depending on number of hidden layers and soft margin parameter

**Figure 3:**
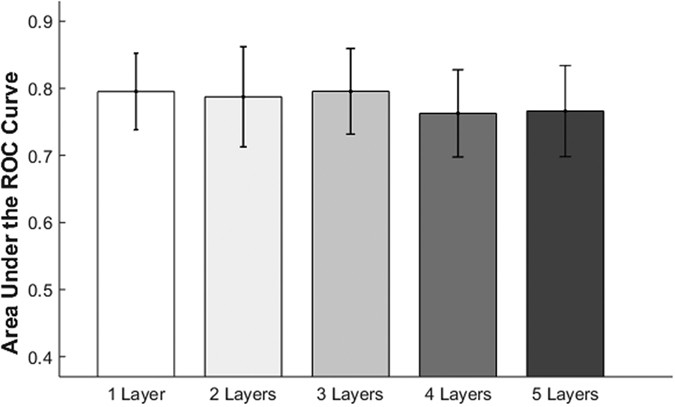
Comparison of the area under the receiver operating characteristic curve (AUC-ROC). The AUC-ROCs of the validation set are shown for deep learning models with one, two, three, four, or five hidden layers.

Figure 3 depicts the average AUC-ROC obtained from the five randomly permuted sets from the cross validation process for the DBN-DNNs with several numbers of hidden layers. The mean and standard deviation of the AUC-ROC of the DBN-DNN with one hidden layers was 0.7953 ± 0.0570, the network with two layers had an AUC-ROC = 0.7875 ± 0.0747, the network with five layers had an AUC-ROC = 0.7661 ± 0.0681, and the network with four hidden layers (0.7628 ± 0.0651) had the lowest score. The DNN with three hidden layers achieved the optimal average AUC-ROC of 0.7957 ± 0.0639.

The performance of the SVM during its optimisation process was the same along all C parameter values investigated during the grid search process (AUC-ROC = 0.7700 ± 0.0773). This result occurred because the SVM model correctly classified all examples from the training set. Thus, we adopted the default value (C = 1) for further analysis. More detailed information about the classifier performance during this search is provided in the supplementary information.

The Bayesian optimisation (with 2000 iterations) replicated seventy-five times (3-fold cross validation replicated five times for 5 different number of hidden layers) took ~56 hours on a system with two Intel(R) Xeon(R) CPU E5-2650 v3 running at 2.3 GHz.

### Classifier performance

We subsequently used the 3-hidden layer deep learning models (which demonstrated the optimal number of hidden layers) during the comparison with the SVM. Table 3 demonstrated the mean performance of the classifiers along the five replications. The DBN-DNN performed better in almost all metrics (except the sensitivity) than the SVM in classifying patients and controls using the volume of anatomical structures and cortical thickness data.

### The most distinct features between the SCZ and HC classes

**Figure 4:**
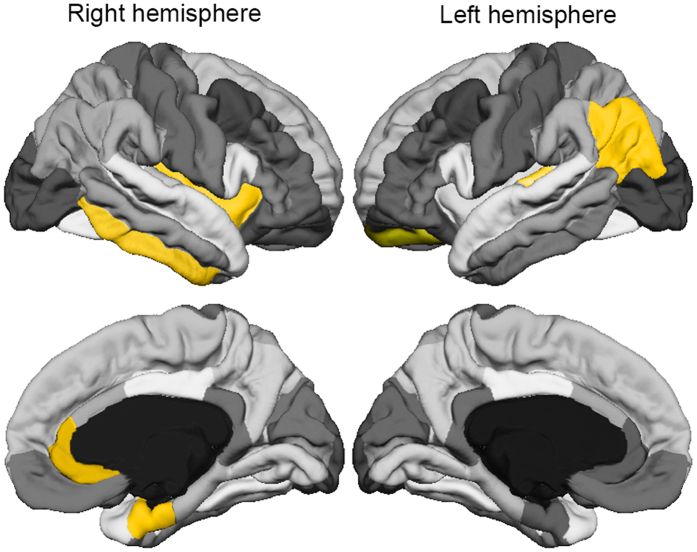
The cortical regions with highest differences between healthy control and schizophrenia groups. These cortical regions (highlighted in yellow), include right entorhinal cortex, right rostral anterior cingulate, left inferior parietal cortex, right insula, right inferior temporal cortex, left lateral orbitofrontal cortex, and left transverse temporal cortex. The different shades of grey represent the parcellation of the Desikan-Killiany atlas.

The best DBN-DNN model with four hidden layers from all fifteen replications (5 times 3-fold cross validation) achieved a AUC-ROC of 0.9132 when applied on the validation set, and this model had 183 units per hidden layer, unsupervised learning rate = 0.05258, and supervised learning rate = 0.06102. We used this model to generate samples for both classes: SCZ and HC. From these samples, we calculated the difference between the resulting input vectors (i.e., brain region data). This approach obtains the most different features across both classes, which are found at the most abstract and invariant layers of the DBN based-model. The ten most different brain measures in descending order were: right cerebellum white matter, right lateral ventricle, right entorhinal cortex, right rostral anterior cingulate, left inferior parietal cortex, right insula, left transverse temporal cortex, right inferior temporal cortex, left lateral orbitofrontal cortex, and left putamen (Fig. 4).

### Classification of patients with first-episode psychosis

Finally, we used the same DBN-DNN models that achieved the best performance classifying between SCZ and HC from the validation set. This model was used to predict the diagnosis of the 32 FEP subjects. During this task, the model classified 56.3 ± 6.79%of the FEP subjects as SCZ.

## Discussion

In this study, a DBN-DNN was trained to classify individuals with SCZ and HC, achieving modestly higher predictive performance than the shallow-architecture SVM approach. As a deep learning model, the DBN automatically learns complex mapping by transforming the neuromorphometric features through multiple layers of nonlinear processing. These transformations created representations at a higher abstract level that were used for the classification task. The deep learning classifier out-performed the widely used linear SVM method. Moreover, it was possible to sample the classes’ representations created by the DBN-based model. From the difference of these samples, we identified brain morphological features that could best differentiate between the classes. Finally, we evaluated the performance of the classifier in predicting the diagnosis of subjects with FEP. This classifier was trained only with data from the other classes, and produced an error rate of 56.3%. This suggests that FEP could represent a third classification in which brain morphology is on a continuum between SCZ and HC morphologies.

Until recently, most machine learning techniques had utilised shallow-structured architectures. These models are demonstrably effective in solving many simple or well–constrained problems. However, some studies have identified several reasons to use deep structures^36^,^37^. Deep models may be more robust in the wide variety of functions that can be parameterized by composing weakly non-linear transformations. They also allow for the representation of highly varying functions more efficiently than do shallow architectures. Moreover, a major issue in conventional techniques is the need for careful engineering and considerable domain expertise, in order to design a feature extractor that transforms the raw data into an appropriate feature vector. Deep learning allows a system input to be compositing from raw data, thus allowing the machine automatically discover the representations required for machine learning tasks^7^,^38^. Finally, the appeal of hierarchical representations, and the potential for combining unsupervised and supervised methods, also contribute to the use of deep architectures^36^. In this study, we did not explore all possible deep learning advantages, like the use of input data without feature extraction. The raw MRI data was not used as input data due the high dimensionality of it (composed of hundreds of thousands of voxels) and of computer resource demand required to train a DBN-based model. Even so, experimental results show that when using structural neuroimaging, the DBN-based model can achieve better differentiation performance than shallow-architecture methods used for comparison.

In analysing the most distinct features between the groups, we applied the sampling process of the conditional distributions of each class that the DBN-DNN learned. We found several brain measures that differed between groups and that have been described in other SCZ morphometric studies^39^,^40^,^41^,^42^,^43^,^44^. The right cerebellar white matter demonstrated the greatest difference between groups. In accordance with this, several studies have noted cerebellar abnormalities in SCZ, suggesting that the brain region is significantly associated with the disorder^39^,^40^. In addition, multiple reports have described decreased cortical thickness in temporal, parietal and frontal cortices in patients diagnosed with SCZ^41^,^42^. The difference in the right insular cortex was also reported by Palaniyappan *et al*.^43^, who suggested it related to the loss of insight observed in SCZ. Enlarged ventricles are one of the most consistently reported brain abnormalities in SCZ^42^. The difference in left putamen volume appears to be related to poorer verbal learning, executive functioning and working memory performance^45^. DBN-based methodology could be used as a new multivariate pattern analysis to reveal the complex associations between the neural substrates mediating SCZ and other psychiatric disorders.

Finally, we analysed the effectiveness of the DBN model in classifying patients with FEP as presumably having schizophrenia (i.e., into the SCZ group). However, FEP was classified incorrectly very near half the time. For this reason, we believe that the DBN deep structure model would consider patients with FEP as a class with brain morphology falling midway between HC and SCZ patterns. The features learned from the SCZ and HC groups were not suitable to define these patients with FEP in a particular class. In general, there is consistency amongst anatomical studies showing that changes in subjects with FEP are less widespread than those in chronic SCZ^46^,^47^. This difference is meaningful enough to affect the accuracy of our classifier, since it was trained using features found only in chronic SCZ.

The main limitation of this study is the relatively small number of training samples. In neuroimaging studies with data from psychiatric patients, it can be difficult to obtain a large number of samples. The recent trend toward increased sharing of neuroimaging data in the research community should increase the available training set for future studies, enhancing the construction of effective models for clinical psychiatry^48^. Public databases, e.g. the Center for Biomedical Research Excellence (COBRE) schizophrenia sample, Northwestern University Schizophrenia Data and Software Tool (NUSDAST; available at http://schizconnect.org/), or the Child and Adolescent NeuroDevelopment Initiative (CANDI) Share Schizophrenia Bulletin 2008 (available at www.nitrc.org/projects/cs_schizbull08/), may improve the results of classifiers using deep learning methods that can capture high-level concepts and nullify the effect of input variations.

During the selection of the optimal DBN-DNN, we noticed that the performance of the network with one layer was very close to the best average performance from the 3-layers DBN-DNN. This finding might indicate a problem of under/overfitting along the different configurations, and it might affect the full potential of the deeper DBN-DNN. We could attenuate the effect of this issue if we used additional regularization methods in our models (like dropout^49^) or techniques that monitor the validation set metrics during the training to find the optimum number of epochs, like early stopping. However, these approaches would increase the time consumed and the number of hyperparameters during the Bayesian optimisation (e.g., the dropout probability of each layer).

The strength of deep architectures is the multiple levels of nonlinear processing that are well-suited to capture highly varying functions with a compact set of parameters. The results of the present study suggest deep architecture provides superior performance in classification tasks. The deep learning models advance in neuroimaging-based prediction methods, and can be useful for demonstrating complex and subtle associations, as well as enabling more accurate individual-level clinical assessments. Furthermore, the technique is not limited to morphometric data from structural MRI; it can be readily applied to data from other modalities, including fMRI and DTI.

## Additional Information

**How to cite this article**: Pinaya, W. H. L. *et al*. Using deep belief network modelling to characterize differences in brain morphometry in schizophrenia. *Sci. Rep.* **6**, 38897; doi: 10.1038/srep38897 (2016).

**Publisher's note:** Springer Nature remains neutral with regard to jurisdictional claims in published maps and institutional affiliations.

## Supplementary Material

Supplementary Information

## Figures and Tables

**Table 1:** Patient demographic and clinical data.

	Patients with schizophrenia (n = 143)	First-episode psychosis (n = 32)	Healthy controls (n = 83)	Statistical tests	
				Statistics	p-value
Age, y (avg ± sd)	37.12 ± 10.99	27.09 ± 7.97	35.49 ± 11.08	t = −1.1	0.272
Age of onset, y (avg ± sd)	22.86 ± 6.64	—	—		
Duration of illness, y(avg ± sd)	13.39 ± 8.20	—	—		
PANSS scores (avg ± sd)					
Total	58.59 ± 14.47	—	—		
Positive	12.74 ± 4.46	—	—		
Negative	16.92 ± 5.50	—	—		
General	29.06 ± 7.35	—	—		
Sex, n (%)				χ^2^ = 0.2	0.598
Men	95 (66%)	15 (46%)	56 (67%)		
Women	48 (34%)	17 (54%)	27 (33%)		

Notes: The chi-square test for independence was applied to verify the independence of the gender variable of patients with schizophrenia and healthy controls, and the independent-samples t-test to verify the independence of the age variable.

**Table 2:** Hyperparameters and their optimisation search space.

Hyperparameter	Distribution	Range
Number of units per hidden layer	Quantized uniform	[10, 200]
Learning rate in RBM unsupervised training	Uniform	[1e-1, 1e-4]
Learning rate of the gradient descent algorithm	Uniform	[1e-1, 1e-4]

Notes: Each search space is composed of the original distribution type and range. These search space values are used in the sampling of hyperparameter values in each optimisation iteration. At the end of the iteration, the distribution is modified according to the classifier performance.

**Table 3:** The performance of classification of patients with schizophrenia and healthy controls from structural MRI scans.

Classifier	Balanced accuracy (%)	Sensitivity (%)	Specificity (%)	Error rate (%)
SVM Linear	68.10 ± 9.39	**77.34 ± 12.3**	58.87 ± 17.26	29.82 ± 8.99
DBN-DNN	**73.55 ± 6.84**	76.37 ± 0.090	**70.74 ± 12.17**	**26.14 ± 6.48**

Boldface type represents the better performance scores.
