# Supplementary material for: Using deep belief network modelling to characterize differences in brain morphometry in schizophrenia
Source: Sci Rep. 2016 Dec 12;6:38897. doi: 10.1038/srep38897 (PMC5151017; doi:10.1038/srep38897)
Supplement: Supplementary Information [file srep38897-s1.pdf]

Using deep belief network modelling to characterize differences in brain morphometry in schizophrenia

Walter H. L. Pinaya * [a]; Ary Gadelha [b]; Orla M. Doyle[c]; Cristiano Noto [b]; André Zugman [d]; Quirino Cordeiro [b, e]; Andrea P. Jackowski [b]; Rodrigo A. Bressan [b]; João R. Sato [a, b]

[* a] Center of Mathematics, Computation, and Cognition. Universidade Federal do ABC, Santo André, Brazil.

[b] Department of Psychiatry. Universidade Federal de São Paulo, São Paulo, Brazil.

[c] Department of Neuroimaging, Institute of Psychiatry, Psychology and Neuroscience, King's College London, London, United Kingdom

[d] Interdisciplinary Lab for Clinical Neurosciences (LiNC), Universidade Federal de Sao Paulo, Sao Paulo, Brazil;

[e] Department of Psychiatry, Faculdade de Ciências Médicas da Santa Casa de São Paulo, São Paulo, Brazil.

[* a] Rua Arcturus, 03 - Jardim Antares, São Bernardo do Campo - SP, CEP 09.606-070, Brazil.

[b] Rua Borges Lagoa, 570 – Vila Clementino, São Paulo - SP, CEP 04.038-020, Brazil.

[c] Institute of Psychiatry (PO89), King's College London, De Crespigny Park, London SE5 8AF, UK

[d] Rua Borges Lagoa, 570 – Vila Clementino, São Paulo – SP, CEP: 04.038-020, Brazil.

[e] Rua Major Maragliano, 241 - Vila Mariana, São Paulo - SP, CEP 04.017-030, Brazil.

Corresponding Author: Walter H. L. Pinaya

Phone: +55 11 97123 0508

Email address: walhugolp@gmail.com

Supplementary information

Deep Belief Networks

The deep learning method that we used in this study consisted of a deep neural network pre-trained by a DBN (DBN-DNN). The DBN has gained popularity since the successful implementation of an efficient learning technique that stacks simpler models known as restricted Boltzmann machine (RBM)[6].

Restricted Boltzmann Machine

The RBM can be interpreted as an artificial neural network that extracts latent features of the input unknown probability distribution based only on observed samples[19]. Given some observations, training an RBM means adjusting the model parameters such that the probability distribution represented by it fits the distribution of the training data as well as possible.

The RBM network consists of a bipartite graph that has a visible layer and a hidden layer (Fig. 1). The RBM can be defined as an energy-based model, and the joint probability distribution of hidden unit values $\mathbf{h}$ and visible unit values $\mathbf{v}$ is determined using an energy function E (1).

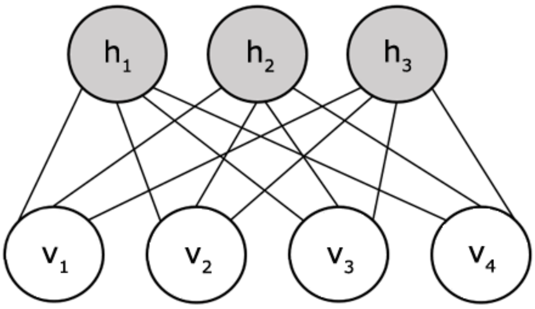

Figure 1. Restricted Boltzmann Machine (RBM). The graph of an RBM has only connections between the layer of hidden (gray circles) and visible variables (white circles) but not between two units

of the same layer. This means that the hidden units are independent of each other given the state of the visible units and vice versa.

$$\mathrm{P}(\mathbf{v},\mathbf{h}) = \frac{1}{Z}\exp(-\mathrm{E}(\mathbf{v},\mathbf{h})) \text{ equation (1)}$$

$$Z = \sum_{\mathbf{v}}\sum_{\mathbf{h}}\exp(-\mathrm{E}(\mathbf{v},\mathbf{h})) \text{ equation (2)}$$

where the normalizing constant Z is called the partition function by analogy with physical systems. The partition function is obtained by summing over all possible pairs of visible and hidden vectors (2).

The RBM hidden units are typically treated as binary stochastic units (with a Bernoulli distribution). The visible layer can also handle binary data distribution with Bernoulli units. However, the RBM can also handle continuous data distribution (like the morphometric data) with visible Gaussian units. These units conditionally follow a Gaussian distribution which mean is determined by the weighted sum of the states of the hidden units. The RBM that uses this type of visible unit is called as Gaussian-Bernoulli RBM (GRBM). The GRBMs can be used to convert real-valued variables of DBN input layer to binary stochastic variables, which can then be treated using the Bernoulli-Bernoulli RBMs. Thus, the energy function in the Bernoulli-Bernoulli RBM is defined by:

$$\mathrm{E}(\mathbf{v},\mathbf{h}) = -\sum_i b_i v_i - \sum_j c_j h_j - \sum_i\sum_j v_i W_{i,j} h_j \text{ equation (3)}$$

The energy function of GRBM can be defined by:

$$E(\mathbf{v},\mathbf{h}) = \frac{1}{2}\sum_i v_i^2 - \sum_i b_i v_i - \sum_j c_j h_j - \sum_i \sum_j v_i W_{i,j} h_j \quad \text{equation (4)}$$

where b_i and c_j are the bias of visible unit i and hidden unit j, respectively, and $W_{i,j}$ is the weight parameter of the model connections.

The objective of training is to fit the probability distribution model over a set of visible random variables $\mathbf{v}$ to the observed data. Thus, the training process can be operated by maximum likelihood estimation method for the marginal probability $P(\mathbf{v}) = \sum_{\mathbf{h}} P(\mathbf{v},\mathbf{h})$. The gradient of the likelihood on the RBM parameters (weights and biases) has a closed form. However, it includes an intractable expectation over the joint distribution of visible and hidden $P(\mathbf{v},\mathbf{h})$.

Usually, an approximation of the gradient is used to deal with this intractable expectation problem. A truncated version of Gibbs sampling method called Contrastive Divergence (CD)[6] uses the conditional probability, $P(\mathbf{v}|\mathbf{h})$ and $P(\mathbf{h}|\mathbf{v})$ in the approximation. The popularity of the RBM stems from CD efficient algorithm and from the ability to calculate conditional distributions over $\mathbf{v}$ and $\mathbf{h}$ easily. The conditional probabilities of the RBM can be computed as:

$$P(h_j = 1 | \mathbf{v}) = \sigma\left(c_j + \sum_i v_i W_{i,j}\right) \quad \text{equation (5)}$$

$$P(v_i = 1 | \mathbf{h}) = \sigma\left(b_i + \sum_j h_j W_{i,j}\right) \quad \text{equation (6)}$$

Similarly, for a GRBM, the corresponding conditional probability of visible units become:

$$\mathrm{P}(v_i = 1 \mid \mathbf{h}) = \mathrm{N}\left(b_i + \sum_j h_j W_{i,j}; 1\right)$$ equation (7)

where σ the logistic sigmoid function ($\sigma(x)=1/(1+e^{-x})$), and the normal distribution is denoted by N(mean;variance). Further information on RBM model and training can be found in [6,19].

Creating Deep Belief Networks

After training, the hidden unit values of RBM provide a closed-form representation of the dependencies between the visible units. The idea is that the hidden units extracted relevant features from the observations. However, these features are regarded as low-level features. To achieve more complex representations, the model needs to calculate the higher-level features based on the lower-level ones. So, we create a DBN by stacking RBMs[6]. The stacking procedure is described as follows. After training a GRBM with the continuous input data, we treat the activation probabilities of its hidden units as the input data to train the Bernoulli–Bernoulli RBM one layer up. Similarly, the hidden units' activation probabilities of the second-layer RBM are used as input for next RBM, and so on until reaching the desired depth. By stacking RBMs, the DBN can learn a hierarchical structure of the input data.

This "pre-training" can be followed by a discriminative training that fine-tunes all layers jointly to perform the classification task. This fine-tuning is done by initiating the parameters of a deep neural network with the values of DBN pre-trained parameters. Besides that, final layer (composed of softmax units) is added to

implement the desired targets of the training data, the labels SCZ and HC. Finally, the backpropagation algorithm and a gradient-based optimization algorithm can be used to adjust the network parameters, creating a DBN-DNN.

Detailed information of the selection of the DBN-DNN optimal models

Table 1 The AUC-ROC of the DBN-DNN classifiers during the search for the optimal number of hidden layers.

#	Cross validation	1 Layer	2 Layers	3 Layers	4 Layers	5 Layers
1	1	0.8697	0.8889	0.8640	0.8649	0.8640
	2	0.8067	0.7858	0.8008	0.8392	0.6892
	3	0.8778	0.8704	0.8269	0.8417	0.8093
2	1	0.7339	0.7688	0.7839	0.6491	0.7304
	2	0.8121	0.8030	0.8924	0.7441	0.8076
	3	0.7294	0.7301	0.6934	0.7902	0.7441
3	1	0.9174	0.9104	0.9132	0.7692	0.9062
	2	0.8269	0.8278	0.8295	0.7631	0.7019
	3	0.7540	0.7692	0.7596	0.7917	0.7628
4	1	0.7738	0.8185	0.7554	0.7900	0.7677
	2	0.7750	0.8033	0.8383	0.7600	0.7875
	3	0.7617	0.7200	0.7258	0.7139	0.7200
5	1	0.7950	0.6091	0.7723	0.6273	0.7662
	2	0.7304	0.7441	0.7308	0.7471	0.7981
	3	0.7662	0.7628	0.7485	0.7500	0.6371
Mean		**0.7953**	**0.7875**	**0.7957**	**0.7628**	**0.7661**
Standard deviation		**0.0570**	**0.0747**	**0.0639**	**0.0651**	**0.0681**
